# The cross-talk between lateral sheet dimensions of pristine graphene oxide nanoparticles and Ni^2+^ adsorption†

**DOI:** 10.1039/d1ra00400j

**Published:** 2021-03-19

**Authors:** Majdoleen Atawneh, Sami Makharza, Sahar Zahran, Kariman Titi, Fahed Takrori, Silke Hampel

**Affiliations:** Faculty of Science and Technology, Department of Chemistry, Hebron University P.O. Box 40 Hebron West Bank Palestine; College of Medicine, Hebron University P.O. Box 40 Hebron West Bank Palestine samim@hebron.edu; IFW Dresden Germany

## Abstract

This study investigated the removal of nickel(ii) ions by using two sizes of graphene oxide nanoparticles (GO – 450 nm and GO – 200 nm). The thickness and lateral sheet dimensions of GO are considered to be an important adsorbent and promising method for sufficient removal of metals like nickel, lead, copper, *etc.* The graphite oxide was prepared by oxidation–reduction reaction (Hummers method), and the final product was labelled as GO – 450 nm. A tip sonicator was used to reduce the size of particles to 200 nm under controlled conditions (time and power of sonication). FTIR spectroscopy shows that both sizes of GO particles contain several types of oxygen groups distributed onto the surface of GO particles. Scanning electron microscopy (SEM) and the statistical analysis confirmed the formation of these two sizes of GO particles. The GO – 200 nm performed better removal of Ni(ii) compared with GO – 450 nm, due to more surfaces being available. The adsorption capacity of GO particles increased drastically from 45 mg g^−1^ to 75 mg g^−1^ for GO – 450 nm and GO – 200 nm respectively, these values were carried out after 2 h of incubation. The kinetics of adsorption and several parameters like initial concentration at equilibrium, pH, temperature, and adsorbent dose are controlled and studied by using UV-visible spectroscopy. The results indicated a significant potential of GO – 200 nm as an adsorbent for Ni(ii) ion removal. An additional experiment was performed to estimate the surface area of GO – 450 nm and GO – 200 nm, the results show that the surface areas of GO – 450 nm and GO – 200 nm are 747.8 m^2^ g^−1^ and 1052.2 m^2^ g^−1^ respectively.

## Abstract 2

This study investigated the removal of nickel(ii) ions by using two sizes of graphene oxide nanoparticles (GO – 450 nm and GO – 200 nm).

## Graphical abstract

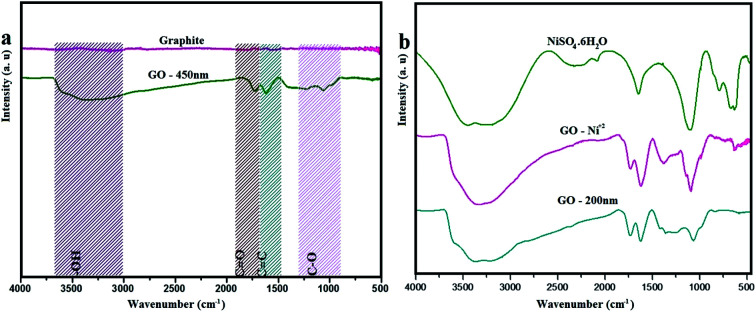

## Introduction

Graphene oxide (GO) has been investigated as an extraordinary next-generation adsorbent in water and wastewater purification.^1–3^ The neat structure and outstanding physicochemical characteristics of GO have attracted enormous attention from researchers for use in several fields of technology, such as energy, biology, medicine, and water.^2,4–14^ GO is a two-dimensional layer of sp^2^ hybridized carbon atoms decorated with abundant oxygen groups like carboxyl, hydroxyl, epoxy, *etc.*, these groups have the ability to bind with organic and inorganic substances with both chemical and/or physical interactions. Moreover due to its highly hydrophilic surfaces, open flake morphology and high adsorption capacity towards different metallic ions, organic and inorganic materials, several reports have been focused on GO as a promising nanosystem in wastewater treatment. It exhibits no obvious toxicity under low dose (*ca.* 0.2 mg) and medium dose (*ca.* 0.2–0.25 mg).^15–17^

Heavy metals such as cadmium, cobalt, lead, nickel are non-bio-degradable and cause various diseases and disorders. Nickel is a transition metal ion that has four oxidation states (+1, +2, +3, and +4), it is widely used in industry such as mining, smelting, textiles, fertilizer, electroplating, battery manufacturing, and pigment production. At high concentrations, Ni^2+^ may lead to stern damage of vital organs such as lungs, kidneys, gastrointestinal irritation, and lung and bone cancers. The Ni^2+^ in wastewater varies from a low value of 0.5 mg l^−1^ to a high value of 1000 mg l^−1^. The maximum permissible safe limit of Ni^2+^ with industrial effluents into land water is 3 mg l.^3,18,19^ Innovative approaches for treating industrial wastewater including heavy metals often involve new technologies for reducing poisonousin order to gather technology-based treatment standards. The incorporation of nanoparticles such as functionalized graphene and carbon nanotubes into water purification technologies against the removal of heavy metals appears as a very dynamic branch of nanotechnology. Nanoparticles owe their potential to the high specific surface area and surface reactivity compared to conventional substances. Depending on the mechanism of uptake, nanoparticles can be destined to establish high selectivity against various types of pollutants.Graphene oxide has a very extensive advantage in treating various heavy metal ions from aqueous solutions, such as lead (Pb(ii)),^20,21^ copper (Cu(ii)),^22,23^ cobalt (Co(ii)),^24^ cadmium (Cd(ii)),^22^ chromium (Cr(vi)).^25^ Moreover, GO functionalized by using organic molecules inorder to enhance the surface selectivity of pollutants.^22,26,27^

Nowadays, an extensive research on the facile synthesis of 2D carbon nanomaterials with inorganic substances derived from MOF precursors for various applications.^28–30^ The determination of heavy metal ions in water can be achieved using various methods. The simple and facile method is a chemical reagent method such as (4-(2-pyridylazo) resorcinol) which depends on changes in color when reacting with heavy metal ions. The more precise detection method is carried out using instruments like inductively coupled plasma-mass spectrometry (ICP-MS), atomic absorption spectrophotometer (AAS), and X-ray fluorescence spectrometry (XRF). However, those techniques require additional chemicals, expensive equipment, and trained users. Thus, in this article, ultraviolet/visible (UV/VIS) spectroscopy method was employed as a non-destructive method for the detection of nickel(ii) without chemical reagents.^31^

To the best of our knowledge, no studies have been conducted to investigate the effect of lateral sheet dimensions of pristine GO on the adsorption of Ni^2+^ in solution, as well as no studies joint between the size distribution and surface area of GO nanoparticles. We have investigated two sizes of GO nanoparticles (GO – 450 nm and GO – 200 nm) at different time intervals, concentration, pH value, adsorbent dose and temperature.

## Materials and methods

### Synthesis of graphite oxide

As shown in Scheme 1 (ESI†), graphite oxide was prepared by using the Hummer's method and conventionally by the oxidation–exfoliation reaction.^32–34^ Briefly, 1.0 g of graphite and 50 g NaCl were grounded in mortar for 20 min, the ground graphite was dissolved in distilled water, filtrated, washed several times and dried in an oven at 40 °C for 6 h. The filtrated graphite was stirred in 23 ml 95% H_2_SO_4_ overnight. The mixture was placed in an ice bath (below 10 °C) for starting the oxidation step, 3.0 g KMnO_4_ was added slowly over 3 hours with continuous stirring. Afterward, the mixture was heated up to 35 °C for 30 min and to 50 °C for 45 min respectively. The mixture cooled down then 46 ml of distilled water was slowly added to the solution, the solution temperature was increased to 98–105 °C with stirring for 45 min. A 140 ml distilled water and 10 ml of 32% H_2_O_2_ were added gradually to terminate the reaction. The final product was filtrated and washed 5 times with 5% HCl and distilled water. Lastly, the graphite oxide was dried in an oven at (50 °C) for 6 h.^34^ The actual yield of GO is 1.46 g.

### Synthesis of graphene oxide nanoparticles

The graphene oxide nanoparticles were prepared according to our previous protocol.^34^ Shortly, 1.0 mg ml^−1^ of graphite oxide was sonicated in an ultra-sonication bath under controlled conditions (power, concentration and time), as shown in Scheme 1,† step no. 3 (Size reduction).

### Preparation of different nickel(ii) concentrations

A 1000 mg l^−1^ stock solution of NiSO_4_·6H_2_O was prepared by dissolving 0.4478 g in 100 ml distilled water. The stock solution was used for the preparation of diluted solutions (200, 300, 400, 500, 600, 700, 800, and 900 ppm).

### Batch adsorption experiments

Batch adsorption experiments were carried out in a water bath sonicator. The effect of adsorbent dose, contact time, initial concentration, pH, and temperature were studied by using a UV-Vis spectrophotometer. Two sizes of GO particles (450 nm and 200 nm) were used as an adsorbent to understand the cross-talk between different sizes of GO and Ni^2+^ adsorption. A 3.0 ml nickel solution of different concentrations was shaken in closed bottles with 20 mg GO as the desired adsorbent dose for various contact time (10–120 min) and pH values from 2 to 10. The separation of the solid phase from a solution was done by using suction filtration, and then the residual nickel amount in the filtrate was determined.

The surface area of GO nanoparticles was determined by using methylene blue (MB) experiment.^35^ A 100 ppm of methylene blue dye as stock solution was prepared in ultrahigh distilled water, different solutions of methylene blue were prepared for establishing the calibration curve. In separate experiments, 5 mg of GO was mixed with 25 ml of 16, 8, 4, 2 and 1 ppm of MB for 10 min at 20 °C and 4000 rpm. The samples were filtrated and the concentration of MB at equilibrium was calculated.

## Result and discussion

### Scanning electron microscopy (SEM)

The scanning electron microscopy (SEM) is employed to study the morphology and the lateral sizes of GO nanoparticles.^34,36^ As shown in Fig. 2, SEM exhibits two sizes of graphene oxide nanoparticles; as shown in panel (a) the average size (lateral width) of as-prepared GO particles is approximately 450 nm. The average size of GO particles after sonication by using tip sonicator is approximately 200 nm as shown in panel (b). Fig. 2c represents the statistical analysis of GO particles deduced from SEM images. The SEM images were obtained using a FEI, NOVA NanoSEM-200 with an acceleration voltage of 15 kV.

**Fig. 1:**
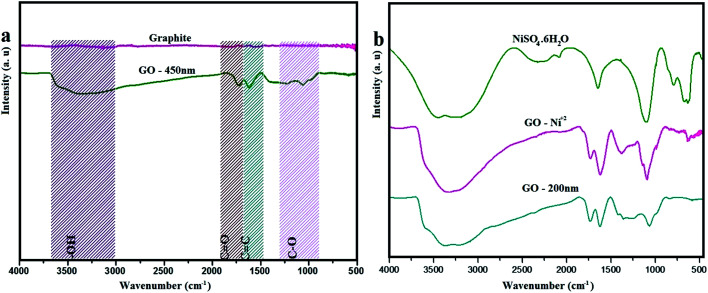
FTIR spectra of (a) graphite and GO – 450 nm. (b) NiSO_4_·6H_2_O, GO – Ni^2+^ and GO – 200 nm.

**Fig. 2:**
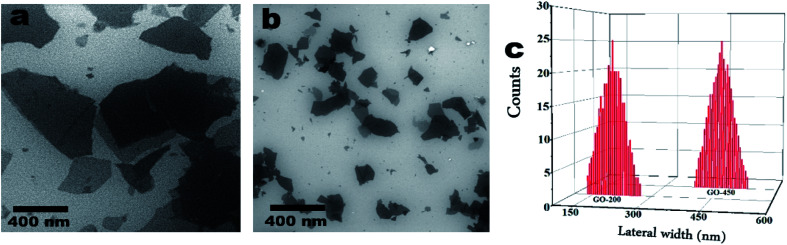
SEM images of (a) GO – 450 nm and (b) 200 nm. Panel (c) is the average width (nm) of GO particles deduced from SEM images.

### FTIR

Fig. 1 exhibits the FTIR spectra of graphite and as prepared graphite oxide (GO – 450 nm). The graphite spectrum shows no notable bands in the region of IR from 4000–500 cm^−1^. However GO reveals variation bands at 3312, 1730, 1612, 1231, 1077 cm^−1^ which corresponding to hydroxyl, carbonyl, –C=C–, epoxy, and C–O groups respectively.^2^ The broad band at 3312 cm^−1^ attributed to the stretching vibration of (–OH) group, which is due to the hydroxyl group of carboxylic acid at the edges of GO sheets as well as the alcohol groups distributed on the basal plane of graphene oxide layers. The significant peak at 1612 cm^−1^ascribed to sp^2^ aromatic pattern (–C=C–), and the peak appeared at 1730 cm^−1^ is due to (–C=O) group. The weak band in the region of 1231 cm^−1^ is assigned to stretching vibration of epoxide group (–C–O), and the peak at 1077 cm^−1^ due to the alkoxy group (–C–O).^24,25^Fig. 1b shows the FTIR spectra of free Ni^2+^, GO – Ni^2+^, and GO – 200 nm. Ni^2+^ reveals a broad peak at 3200 cm^−1^, which assigned to symmetric stretching of the water molecule, moreover the bending vibration of water molecules observed at 1656 cm^−1^. The other peaks at 1096 cm^−1^, 465 cm^−1^, 632 cm^−1^, and 797 cm^−1^ are corresponding to fundamental vibrations of SO_4_^2−^ free ion namely as a non-degenerate, doubly degenerate, triply degenerate mode respectively. After adsorption of Ni^2+^, a broad band at 3312 cm^−1^ in GO was shifted to 3150 cm^−1^. The strong peak at 1730 cm^−1^ shifted to 1715 cm^−1^, which is assigned to Ni^2+^ adsorbed on GO particles. These findings are in agreement with previously reported data.^37,38^ The FTIR spectra were obtained using Bruker FTIR in the range between 4000–500 cm^−1^.

### UV-visible spectrophotometry

The UV-visible Spectrophotometry (UV-Visible line 9100, photometric range: 320–1100 nm, Aqualabo company) was used in this study. Fig. 1a (ESI†) reveals the UV visible spectroscopy of free Ni^2+^ ions at different concentrations. As the Ni^2+^ concentration increases the absorbance increases at different wavelengths (393 nm, 656 nm, and 720 nm). These wavelengths are assigned to *υ*_1_ = ^3^A_2_g (F) → ^1^Eg, *υ*_2_ = ^3^A_2_g (F) → ^3^T_1_g (F), *υ*_3_ = ^3^A_2_g (F) → ^3^T_1_g (P). The observed bands of 393 nm and 720 nm related to *υ*_3_ and *υ*_2_ respectively. While *υ*_1_ refers to 656 nm.^39^ Panel (b) reveals the calibration curve for determining the Ni^2+^ concentrations at equilibrium.

### Adsorption capacity

The adsorption capacity (mg g^−1^) was estimated by using the following eqn (1):1
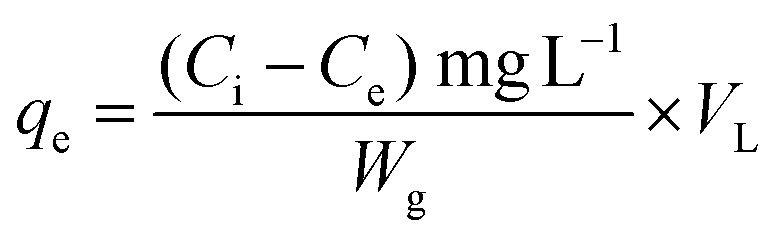
where: *C*_i_ is the initial concentration of Ni^2+^ (mg L^−1^), *C*_e_ is the concentration at equilibrium (mg L^−1^), *W*_g_ is the mass of GO (g), *V*_L_ is the volume of sample.

The adsorption capacity (*q*_e_, mg g^−1^) at different time intervals is increased with increasing the initial Ni^2+^ concentrations as shown in Fig. 4. Moreover, the lateral width of GO particles exhibits a major change in the adsorption capacity due to more surfaces of GO – 200 nm are available. As shown in Fig. 4a, the adsorption capacity of GO – 450 nm appeared as stabilized behavior above 400 mg l^−1^ of Ni^2+^ concentration. In panel b, the GO – 200 nm exhibits a linear behavior with increasing Ni^2+^ concentration up to 900 mg l^−1^ after 60 min of incubation.

**Fig. 3:**
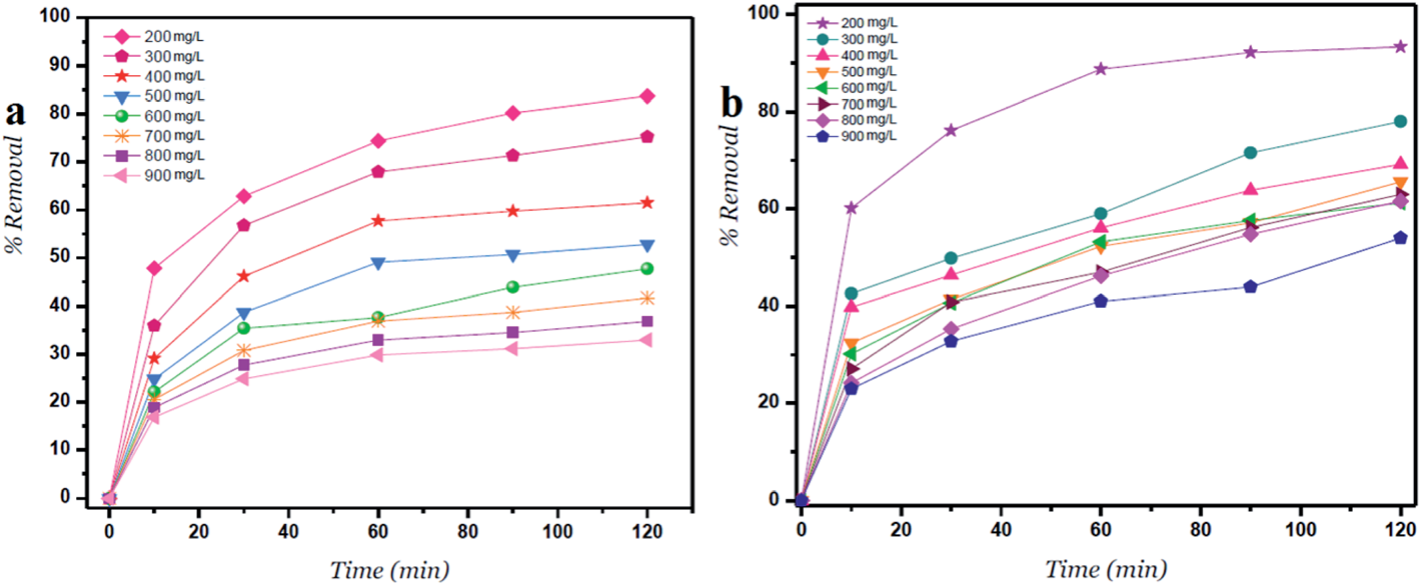
Percent removal *versus* time for (a) GO – 450 nm and (b) GO – 200 nm at different concentrations.

**Fig. 4:**
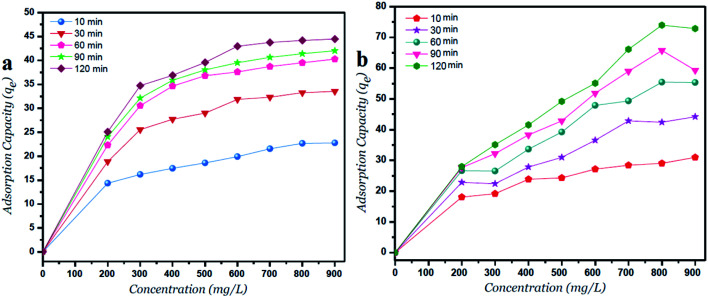
Adsorption capacity *versus* Ni^2+^ concentrations for (a) GO – 450 nm and (b) GO – 200 nm at different time intervals.

### Percent removal of Ni^2+^ at different concentrations and time intervals

Fig. 3 shows the percent removal of Ni^2+^ ions, it depends on the lateral width of GO particles. As the size of GO decreases the percent removal increases due to high surface areas of GO – 200 nm. As shown in panel (a) the contact time reveals a minor change on the removal of Ni^2+^ after 60 min of incubation. In panel (b) the size of GO particles (GO – 200 nm) shows higher removal of Ni^2+^ due to increasing adsorbate surfaces.^1,24^

The removal of Ni^2+^ percent was calculated by using the following eqn (2):2
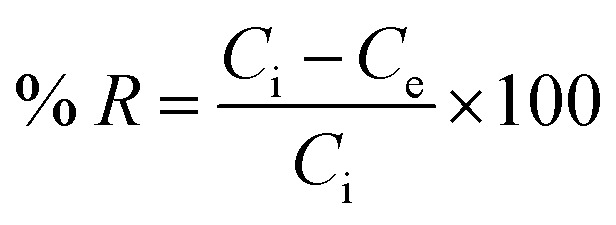
where: *C*_i_ (mg l^−1^) is the initial concentration of Ni^2+^, *C*_e_ (mg l^−1^) is the concentration at equilibrium deduced from the calibration curve.^40^

### Percent removal of Ni^2+^ at different pH

**Fig. 5:**
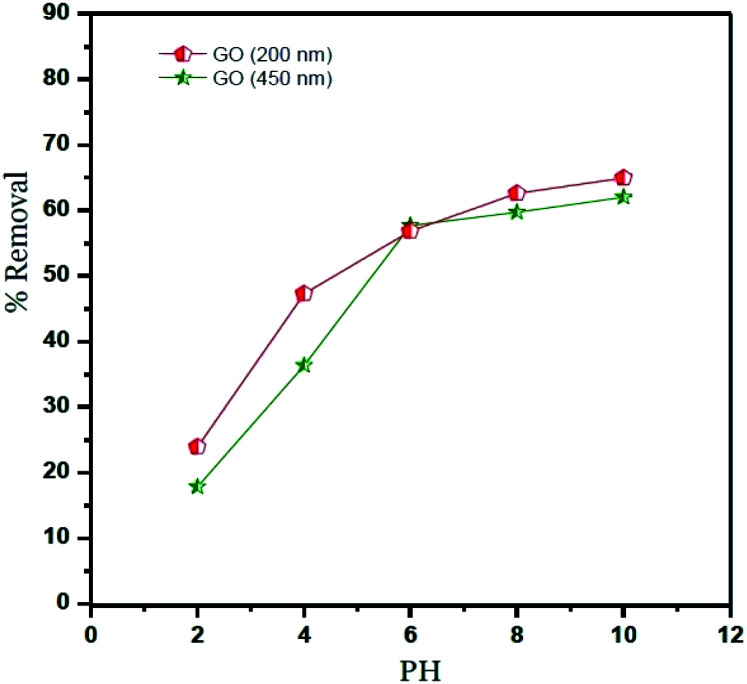
Percent removal of Ni^2+^ at different pH values. Ni^2+^ = 400 mg l^−1^, *T* = 25 °C.

The percentage removal of Ni^2+^ at different pH values from 2 to 10 is shown in Fig. 5, this figure shows that the Ni^2+^ uptake was increased between 2 and 6 pH values. This indicates that the competitive adsorption between Ni^2+^ ions and H_3_O^+^ ions on the surface of GO. At a pH higher than 6, the negative charge on the surface of GO particles increases and lead to strong electrostatic interaction between Ni^2+^ and GO particles under alkaline condition.^18,24^ On the other hand, Ni^2+^ ions can present as (Ni(OH)^+^, Ni(OH)_2_, Ni(OH)_3_^−^,Ni(OH)_4_^2−^) and may be precipitated onto the surface of GO particles.^1,38^

The lateral sheet dimension (GO – 200 nm) shows higher removal comparing with GO – 450 nm. This behavior is attributed to more surfaces of GO particles. In conclusion, the optimum pH value of Ni^2+^ adsorbed onto GO – 450 and GO – 200 nm is 6, which was used for further studies.

### Adsorbent dose

**Fig. 6:**
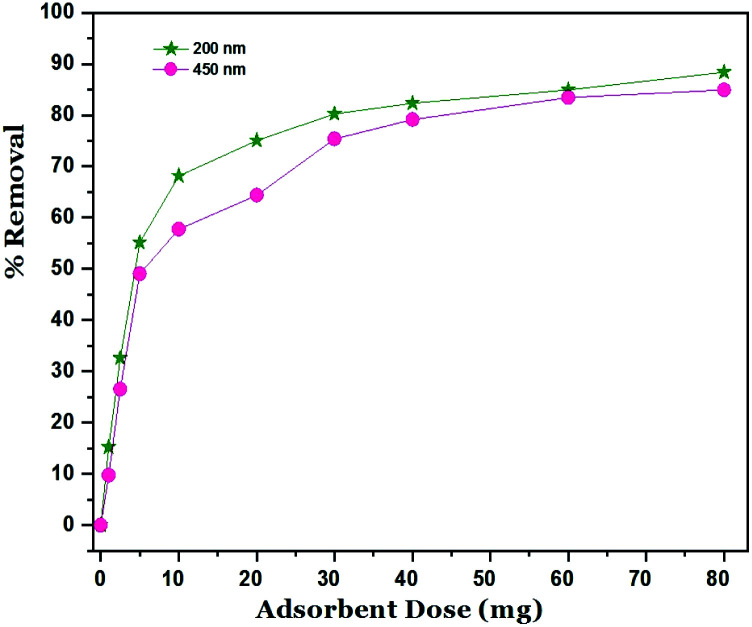
Effect of adsorbent dose (Ni^2+^ = 400 mg l^−1^, *T* = 25 °C, pH = 6).

The effect of adsorbent dose on Ni^2+^ removals is shown in Fig. 6. The amount of GO varying from 1.0 to 80 mg and the initial Ni^2+^ concentration for adsorbent dose was fixed at 400 mg l^−1^. The results reveal that the removal of Ni^2+^ by using GO – 450 nm and GO – 200 nm increased with an increasing of adsorption dose from 1.0 mg to 20 mg, this behaviour is attributed to the increasing of the availability of surfaces at a higher amount of adsorbents. A plateau was reached from 20 up to 80 mg of adsorbent, and this may be imputed to overcrowding in the adsorbed molecules.^1^

### Adsorption isotherm models

The adsorption isotherm plays a key role for the analysis of adsorption process that elucidates how the Ni^2+^ ions distribute between the solid–liquid interface once the system is at equilibrium. The most model used is Langmuir isotherm for monolayer adsorption onto the adsorbent particles. It can be expressed as eqn (3).3
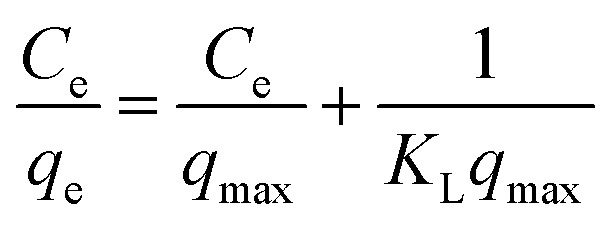
where: *q*_e_ is the amount of Ni^2+^ ions adsorbed per unit weight of adsorbent (mg g^−1^), *C*_e_ is the Ni^2+^ concentration at equilibrium (mg l^−1^). *q*_max_ is the maximum sorption capacity specified by the reactive species in an ideal monolayer system (mg g^−1^). *K*_L_ is the Langmuir constant affined to a free energy of adsorption (l mg^−1^).

The Langmuir parameter (*R*_L_) is a dimensionless constant separation factor defined as in eqn (4)4
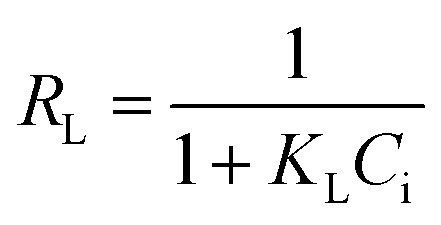
where: *C*_i_ is the maximum initial concentration of adsorbate (mg l^−1^), *K*_L_ is the Langmuir constant (L mg^−1^). *R*_L_ value indicates the adsorption reliable if *R*_L_ > 1 unfavorable, 0 < *R*_L_ < 1 favorable, *R*_L_ = 1 for linear adsorption, and *R*_L_ = 0 for irreversible adsorption.^1^

The Freundlich model is an empirical equation consider that adsorption of metal ions occurs on heterogeneous surface by multilayer adsorption; the linear form of Freundlich can be expressed in eqn (5).5
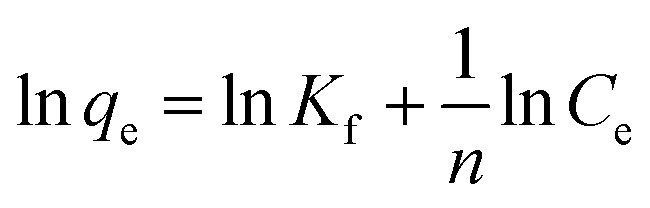
where:*K*_f_ and (*n*) are the Freundlich parameters related to adsorption capacity and adsorption intensity respectively.^41^

### Langmuir and Freundlich isotherm models using GO – 450 nm

**Fig. 7:**
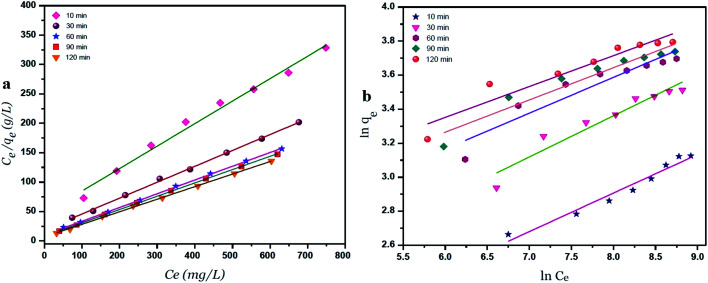
Langmuir (a) and Freundlich (b) adsorption isotherm of Ni^2+^ onto GO – 450 nm at 25 °C.

A plot of *C*_e_/*q*_e_*versus C*_e_ at different time intervals gives a linear relation with the slop of 1/*q*_max_ and intercept of 1/(*K*_L._*q*_max_) as shown in Fig. 7a. The Langmuir model postulates that the uptake of Ni^2+^ ions occurs on a homogenous surface by monolayer adsorption without interaction between the adsorbed ions. For data collection at equilibrium, initial metal concentrations were varied while the GO adsorbent mass remained constant. The adsorption parameters of the Langmuir isotherm model are shown in Table 1 (ESI†) at different time intervals. The correlation coefficient of Langmuir isotherm (*R*^2^) was approximately 0.999, which reveals a high correlation or linear relationship. Therefore the adsorption of Ni^2+^ onto GO – 450 nm is correlated well with the Langmuir equation.

The Freundlich isotherm model interpreted as sorption to heterogeneous surfaces as shown in Fig. 7b. The Freundlich constant *n* between 1 and 10 provides favourable adsorption tends. The larger value of *n* means stronger interaction between the Ni^2+^ ions and GO nanoparticles as shown in Table 1 (ESI†). The Freundlich isotherm parameter (*K*_F_) was calculated according to eqn (5), the value increases as the time interval increased which indicates the Freundlich model fitted well with the experimental data. The correlation coefficient of Freundlich isotherm (*R*^2^) was approximately 0.910.

### Langmuir and Freundlich isotherm models using GO – 200 nm

**Fig. 8:**
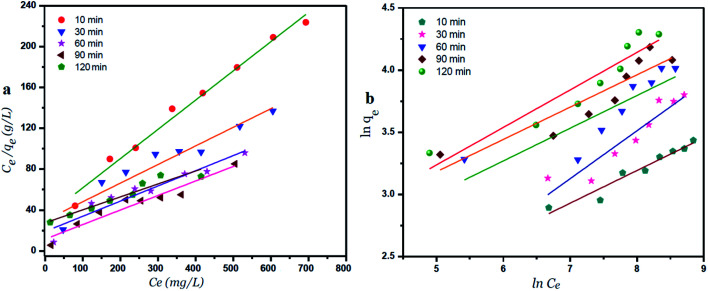
Langmuir (a) and Freundlich (b) adsorption isotherm of Ni^2+^ onto GO – 200 nm at 25 °C.

Fig. 8 exhibits the Langmuir and Freundlich adsorption isotherm of Ni^2+^ onto GO – 200 nm at 25 °C. Panel (a) shows the linear relation between *C*_e_/*q*_e_*versus* Ni^2+^ concentrations at equilibrium for different time intervals. The values of *K*_L_ and *q*_m_ calculated from the plotted data (slope and intercept) are reported in Table 2 (ESI†). The value of *q*_m_ of Ni^2+^ onto GO – 200 nm is higher than that onto GO – 450 nm. This result indicated that a complete and uniform monolayer of Ni^2+^ covering the surfaces of GO particles over the whole concentrations. However the *R*^2^ values at different time intervals were less than that onto GO – 450 nm, indicating that the size of GO – 200 nm particles affect Ni^2+^ uptake onto the adsorbent. Langmuir model indicated the best fitting (*R*^2^ = 0.999) to the isotherm data is with GO – 450 nm size distributions.

In panel (b), the same linear relation appeared between ln *q*_e_*versus* ln *C*_e_ for Freundlich isotherm. The value of *n* is higher than 1 indicated that the adsorption capacity was slightly restrained at lower equilibrium concentration. As shown in Table 2 (ESI†), the values of *n* are studied as an indication of the linearity deviation, as well as it is used to predict the heterogeneity degree of the adsorbent. Moreover the value of *n* is considered as an indication whether the adsorption process is favorable or not. The value of *n* corresponding to GO – 450 nm is greater than that with GO – 200 nm, illustrating that a stronger capacity of Ni^2+^ onto GO – 450 nm. The Freundlich constant *K*_F_ has been found as a relative measure of adsorption capacity. As the value of *K*_F_ increases the adsorption capacity increases. The reported values of *K*_F_ by using GO – 450 nm as adsorbent were greater than that using GO – 200 nm. This result summarizing that the uptake of Ni^2+^ ions with high adsorptive capacity of adsorbent.

**Fig. 9:**
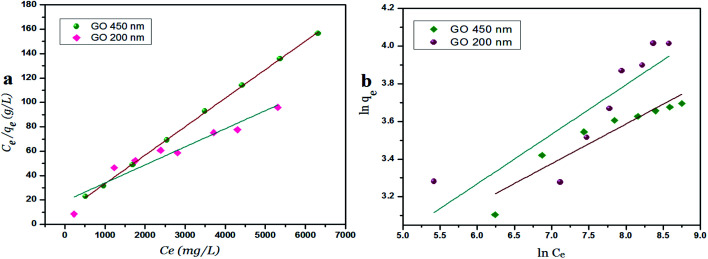
Langmuir (a) and Freundlich (b) adsorption isotherm of Ni^2+^ onto 450 nm and 200 nm at 60 min of incubation.

In this work, the data of Ni^2+^ onto GO – 450 nm and GO – 200 nm of equilibrium adsorption was better fitted to Langmuir than Freundlich adsorption isotherm model. The maximum adsorption capacities of monolayer were 43.478 mg g^−1^ and 66.667 mg g^−1^ for GO – 450 nm and GO – 200 nm respectively as shown in Fig. 9.

The isotherm parameters are summarized in Table 1.

**Table 1:** Langmuir and Freundlich isotherm parameters

Adsorbents	Langmuir			Freundlich	
	*q*_m_	*k*_l_	*R*_L_	*n*	*K*_f_
GO 450 nm	43.478	0.0023	0.046	4.762	6.686
GO 200 nm	66.667	0.0008	0.124	3.802	5.419

### Kinetic study

Two kinetic models were used to investigate the adsorption of Ni^2+^ ions onto GO particles. The models were considered as follows:

Lagergren's pseudo-first-order model which can be expressed by eqn (6):6
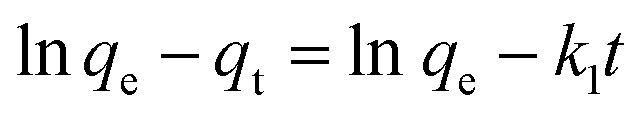
where: *q*_e_ is the adsorption capacity at equilibrium (mg g^−1^), *q*_*t*_ is the adsorption capacity at any time *t* (min). *k*_1_ is the first-order rate constant adsorption min^−1^. Values of *q*_e_ and *k*_1_ were calculated by plotting of ln *q*_e_ − *q*_*t*_*versus* time.

Ho's pseudo-second-order model is given by eqn (7):7
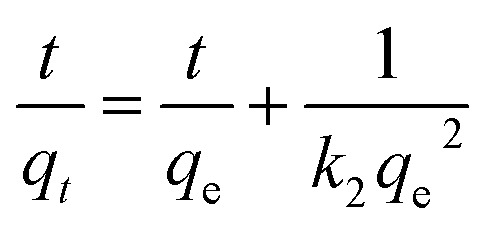
where: *k*_2_ is the rate constant for the pseudo-second-order adsorption (g mg^−1^ min^−1^), values of *k*_2_ and *q*_e_ for different initial concentrations of ions were calculated from the intercept and slope respectively.^42^

**Fig. 10:**
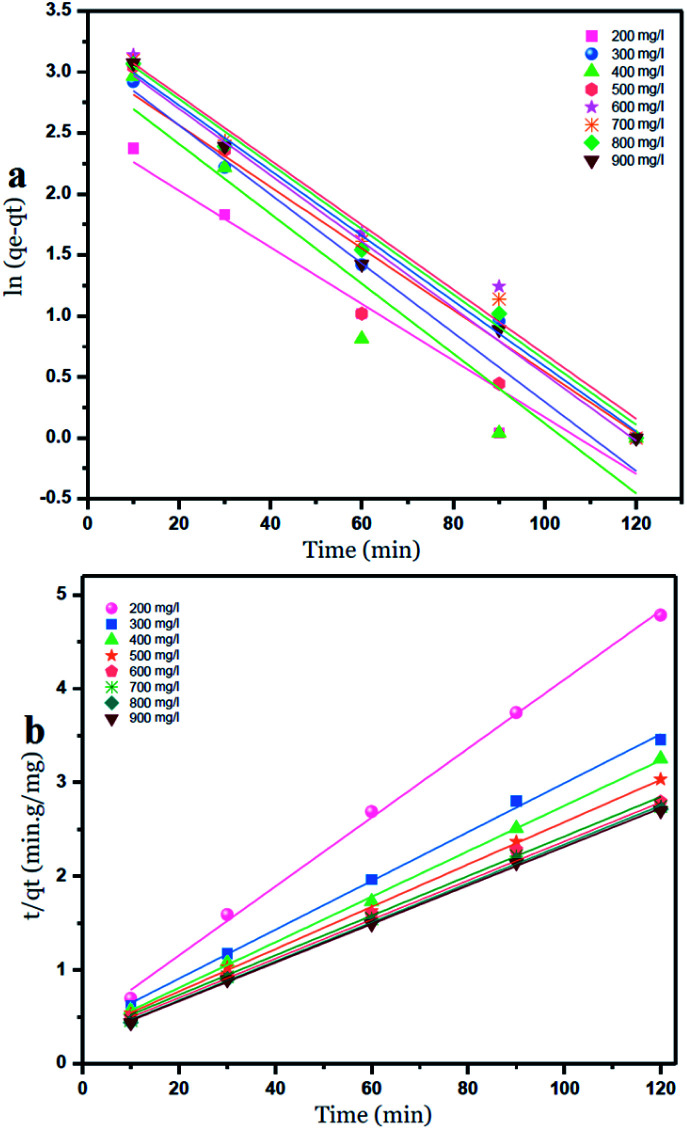
Pseudo first order (a) and Pseudo second order kinetic adsorption model of Ni^2+^ onto GO – 450 nm at 25 °C.

**Fig. 11:**
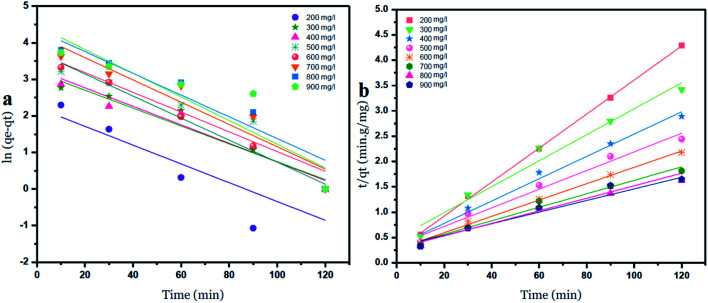
Pseudo first order (a) and Pseudo second order (b) kinetic adsorption model of Ni^2+^ onto GO – 200 nm at 25 °C.

The intercepts and slope of the linear plots for the adsorption of nickel by GO – 450 nm and GO – 200 nm were used to calculate the kinetic parameters, as shown in Fig. 10 and 11, the kinetic constant values are summarized in Table 6 (ESI†).

### Effect of temperature

The adsorption of Ni^2+^ ions was studied at different temperatures (25 °C, 45 °C, and 65 °C), the adsorption data were carried out with 400 mg l^−1^ of Ni^2+^ at pH = 6.

The parameters of thermodynamic like entropy Δ*S*°, enthalpy Δ*H*, and Gibbs free energy Δ*G* can be expressed by Van't Hoff equation8
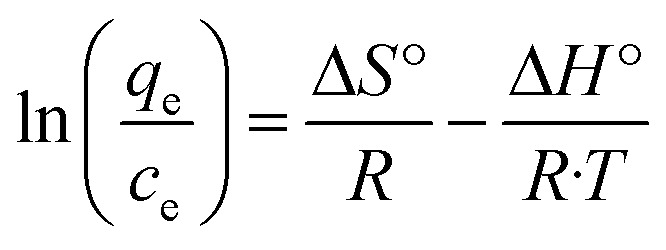
where: *T* (K) is the solution temperature. *R* is the gas constant (8.314 J mol^−1^ K^−1^)

**Fig. 12:**
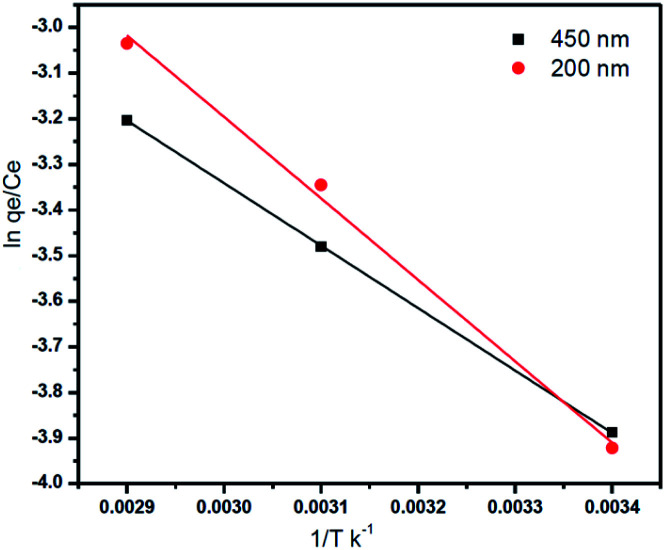
Vant Hoff for the adsorption of Ni^2+^ onto GO – 450 nm and GO – 200 nm. (Ni^2+^ = 400 mg l^−1^).

From the plotting of ln(*q*_e_/C_e_) *versus* (1/*T*) entropy (Δ*S*°) and enthalpy (Δ*H*°)were calculated by using the intercept and slope respectively as shown in Fig. 12, and the Gibbs free energy was calculated by using this eqn (8).^18,43^

As shown in Table 9 (ESI†), the calculated values of the thermodynamic parameters are elucidated. The positive values of Δ*H*°indicate that the adsorption process of Ni^2+^ onto GO – 450 nm and 200 nm is an endothermic process while the positive values of Δ*S*° indicate that increased the randomness at the solid/liquid interface during the adsorption process. The negative values of Δ*G*° suggest that the adsorption of Ni^2+^ onto GO – 450 nm and 200 nm is a spontaneous process but as these values are in the range between −20 and 0 kJ mol^−1^ the process is classified physisorption.^1,18^

### Surface area measurements of GO materials

The specific surface area of graphene oxide (450 nm, 200 nm) was calculated by the following equation:9
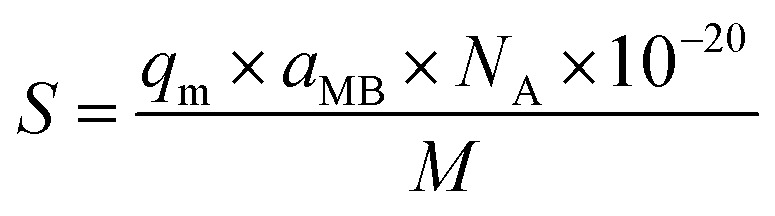
where: *S* is the specific surface area in m^2^ g^−1^; *q*_m_ is the maximum adsorption capacity of methylene blue at the monolayer of GO in mg g^−1^, *a*_MB_ is the occupied surface area of one molecule of methylene blue = 130 Å^2^, *N*_A_ represents Avogadro's number; and *M* is the molecular weight of methylene blue, 373.9 g mol^−1^. The maximum adsorption capacity (*q*_m_) of GO – 450 and GO – 200 nm is 357.1 and 502.5 mg g^−1^ respectively which was evaluated from eqn (3).

## Conclusion

The removal of heavy metal ions from aqueous solution can be achieved by using graphene oxide particles at different size distributions (GO – 450 nm and GO – 200 nm). The smaller size of GO particles provided better removal due to high surface to volume ratio, as well as various oxygen groups like hydroxyl, epoxy and carboxyl present after oxidation and ultrasonication. The adsorption capacity of GO – 450 nm appeared as stabilized behavior above 400 mg l^−1^ Ni^2+^ concentration. However the GO – 200 nm exhibits a linear behavior with increasing Ni^2+^ concentration upto 900 mg l^−1^ after 60 min of incubation. This research demonstrates that GO particles can be an effective adsorbent for toxic metal removal depending on the particle's size distribution.

## Fund

Support fund for Palestinian Universities (Jordan University).

## Conflicts of interest

The authors declare that no competing interests.

## Supplementary Material

RA-011-D1RA00400J-s001
