# Supplementary material for: The cross-talk between lateral sheet dimensions of pristine graphene oxide nanoparticles and Ni2+ adsorption
Source: RSC Adv. 2021 Mar 19;11(19):11388–97. doi: 10.1039/d1ra00400j (PMC8695912; doi:10.1039/d1ra00400j)
Supplement: RA-011-D1RA00400J-s001 [file RA-011-D1RA00400J-s001.pdf]

Electronic Supplementary Material (ESI) for RSC Advances.
This journal is © The Royal Society of Chemistry 2021

Supplementary Information

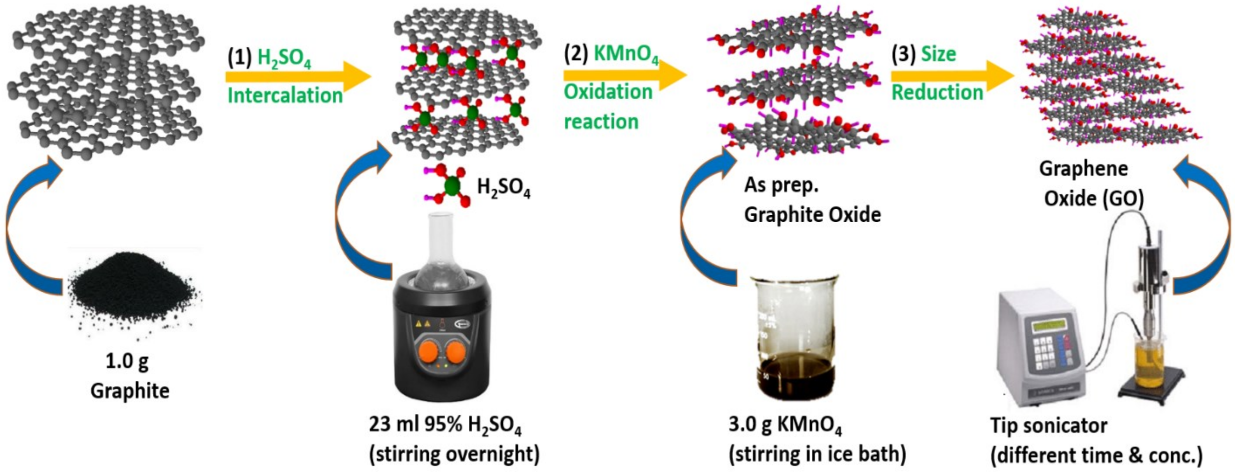

Scheme 1: Schematic representation of graphene oxide formation by using tip sonicator.

Table 1: Langmuir and Freundlich parameters for the adsorption of Ni^{+2} onto GO – 450 nm at 25^0C.

Time (min)	Langmuir isotherm			Freundlich isotherm	
	q_m (mg g^{-1})	K_L (L mg^{-1})	R_L	K_F	n
10	26.316	0.0008	0.122	2.983	4.405
30	37.037	0.0014	0.074	4.149	4.132
60	43.478	0.0023	0.046	6.686	4.762
90	43.487	0.0028	0.036	8.381	5.291
120	46.729	0.0031	0.034	9.593	5.495

Table 2: Langmuir and Freundlich parameters for the adsorption of Ni^{+2} onto GO 200nm at 25^{0}C

Time (min)	Langmuir isotherm			Freundlich isotherm	
	q_m (mg g^{-1})	K_L (L mg^{-1})	R_L	K_F	n
10	34.483	0.0008	0.113	2.968	3.802
30	55.556	0.0006	0.156	1.532	2.591
60	66.667	0.0008	0.124	5.419	3.802
90	71.428	0.0012	0.085	6.567	3.861
120	76.923	0.0005	0.191	5.624	3.331

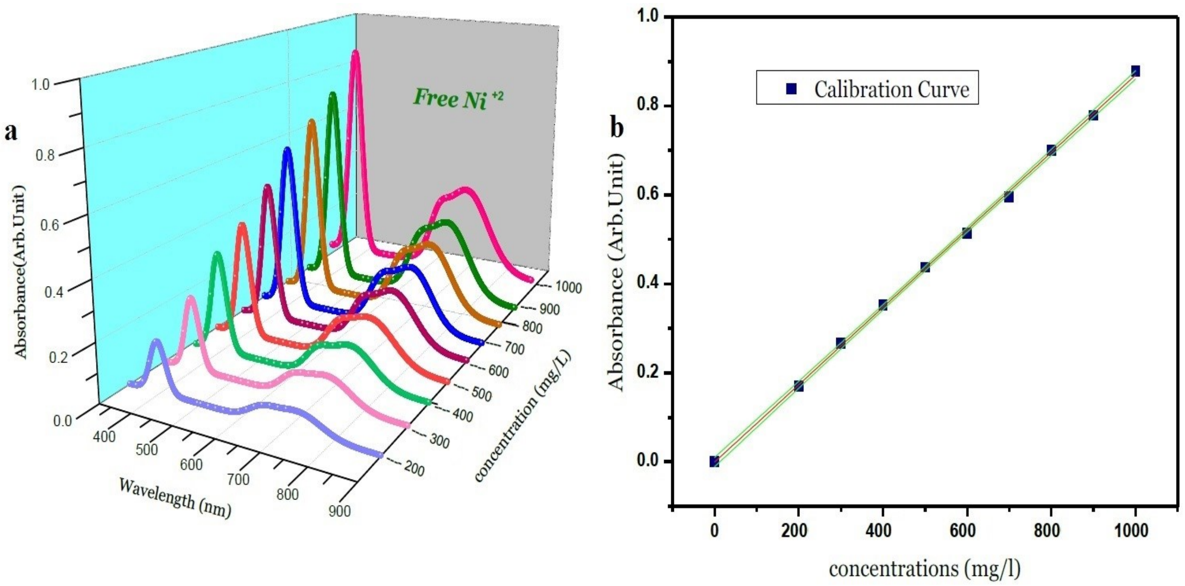

Fig. 1: (a) UV-visible spectroscpy of Ni+2 at different concentrations. (b) The calibration curve of Ni^{+2} samples.

Table 4: pseudo-first-order and pseudo-second-order parameters for the adsorption of the Ni^{+2} onto GO – 450 nm at 25 ^{0}C.

**C_i mg/L**	**$q_{e,exp}$ mg/g**	**Pseudo first order**			**Pseudo second order**		
		**K_1 (min^{-1})**	**$q_{e, cal}$ (mg/g)**	**R^2**	**K_2 g/mg.min**	**$q_{e, cal}$ (mg/g)**	**R^2**
200	25.093	0.023	12.098	0.929	0.00325	27.027	0.998
300	34.718	0.025	21.499	0.983	0.00147	38.462	0.998
400	36.889	0.029	19.747	0.883	0.00177	41.667	0.999
500	39.571	0.028	22.874	0.939	0.00645	43.478	0.999
600	42.963	0.027	28.106	0.968	0.00141	47.619	0.997
700	43.747	0.027	27.495	0.981	0.00153	47.619	0.998

800	44.183	0.028	26.076	0.987	0.00168	47.619	0.998
900	44.447	0.027	25.636	0.987	0.00171	47.619	0.999

Table 5: pseudo-first-order and pseudo-second-order parameters for the adsorption of the Ni^{+2} onto GO – 200 nm at 25 ^{0}C.

C_i mg/L	$q_{e,exp}$ mg/g	Pseudo first order			Pseudo second order		
		K_1 (min^{-1})	$q_{e, cal}$ (mg/g)	R^2	K_2 g/mg.min	$q_{e, cal}$ (mg/g)	R^2
200	27.989	0.026	9.281	0.632	0.00459	29.412	0.999
300	35.105	0.025	26.469	0.930	0.00142	38.462	0.967
400	41.534	0.026	28.588	0.949	0.00143	45.455	0.983
500	49.164	0.027	41.470	0.867	0.00092	55.556	0.976
600	55.079	0.030	42.479	0.989	0.00095	62.500	0.994
700	66.137	0.030	66.819	0.861	0.00057	76.923	0.974
800	73.937	0.032	86.747	0.867	0.00046	83.330	0.971
900	72.825	0.029	77.029	0.741	0.00051	83.330	0.957

Table 6. Kinetic Parameters and regression coefficient (R^2) of Nickel adsorption on GO (450 nm, 200 nm)

Absorbent	$q_{e\ exp\ (mg/g)}$	Pseudo-first-order model			Pseudo-second-order model		
		$K_1(1/min)$	$q_{e\ cal\ (mg/g)}$	R^2	$K_2(1/min)$	$q_{e\ exp\ (mg/g)}$	R^2
GO 450nm	36.889	0.029	19.747	0.883	0.0018	41.667	0.999
GO 200nm	41.534	0.026	28.588	0.940	0.0014	45.455	0.983

Table 7. Comparison of the maximum adsorption capacity $q_m\ (mg/g)$ of several heavy metal ions on GO.

Adsorbent	Metal ions	$q_m (mg/g)$	Conditions	Ref.
GO – 200 nm	Ni^{+2}	66.667	pH=6, T=25 ^{0}C, t=60 min	This study
GO – 450 nm	Ni^{+2}	43.478	pH=6, T=25 ^{0}C, t=60 min	This study
GO	Ni^{+2}	35.6	T= 20 ^{0}C	[1]
GO	Ni^{+2}	38.61	pH=6, T=25 ^{0}C, t=50 min	[39]
GO	Ni^{+2}	20.19	T=25 ^{0}C,	
GO	Cu^{+2}	277.77	pH=6,T= 25 ^{0}C, t=60 min	[40]
GO	Co^{+2}	21.28	pH=5.5,T= 25 ^{0}C, t=60 min	[24]
GO	Pd^{+2}	98.328	T= 25 ^{0}C	[41]

Table 8. Comparison between various adsorbents for removal of Ni^{+2} ions

Adsorbent	Metal ions	$q_m (mg/g)$	Ref.
Fly ash	Ni^{+2}	0.03	[25]
Oxidized CNTs	Ni^{+2}	1.83	[42]
Oxidized MWCNTs	Ni^{+2}	3.73	[43]
Activated carbon prepared from almond husk	Ni^{+2}	30.77-37.18	[44]
Graphene nanosheet/δ-MnO_2 composite	Ni^{+2}	46.55	[45]
Coir pith	Ni^{+2}	9.50	[46]
Carbon aerogel	Ni^{+2}	12.87	[47]
Scrap tire	Ni^{+2}	25.00	[19]
ϒ - Fe_2O_3	Ni^{+2}	23.60	[48]
BT leaf powder	Ni^{+2}	1.527	[49]
Natural zeolite	Ni^{+2}	8.69	[50]

Modified zeolite (NaCl)	Ni^{+2}	10.46	[50]
GO 450 nm	Ni^{+2}	43.347	This study
GO 200 nm	Ni^{+2}	66.67	This study

Table 9: Thermodynamic parameters for the adsorption onto GO-450 nm and GO-200 nm.

Adsorbent	ΔH°	ΔS°	ΔG° kJ/mol		
			25 ^{0}C	45^{0}C	65^{0}C
GO – 450 nm	1.369	6.377	- 0.531	- 0.659	- 0.786
GO – 200 nm	1.786	17.97	- 3.565	- 3.924	- 4.283
